# A systematic review of the literature exploring the interplay between prostate cancer and type two diabetes mellitus

**DOI:** 10.3332/ecancer.2018.802

**Published:** 2018-01-25

**Authors:** Danielle Crawley, Florence Chamberlain, Hans Garmo, Sarah Rudman, Björn Zethelius, Lars Holmberg, Jan Adolfsson, Par Stattin, Paul Carroll, Mieke Van Hemelrijck

**Affiliations:** 1 Translational Oncology and Urology Research Group, King’s College London, London SE1 9RT, UK; 2 Department of Medical Oncology, Guy’s and St Thomas’ NHS Foundation Trust, London SE1 9RT, UK; 3 Department of Public Health and Geriatrics, Uppsala University, Uppsala 751 05, Sweden; 4 Medical Products Agency, Uppsala 751 03, Sweden; 5 Department of Surgical Sciences, Uppsala University, Uppsala 751 05, Sweden; 6 Department of Clinical Science, Intervention and Technology, Karolinska Institutet, Stockholm 171 77, Sweden; 7 Department of Diabetes and Endocrinology, Guy’s and St Thomas’ NHS Foundation Trust, London, SE1 9RT UK

**Keywords:** type two diabetes, prostate cancer, review

## Abstract

Prostate cancer (PCa) and type two diabetes mellitus (T2DM) are both increasing prevalent conditions and often occur concurrently. However, the relationship between the two is more complex than just two prevalent conditions co-existing. This review systematically explores the literature around the interplay between the two conditions. It covers the impact of pre-existing T2DM on PCa incidence, grade and stage, as well as exploring the impact of T2DM on PCa outcomes and mortality and the interaction between T2DM and PCa treatments.

## Introduction

Prostate cancer (PCa) remains the commonest cancer in men, affecting one in eight men in the UK [1]. There are also 3.2 million people in the UK who have been diagnosed with type 2 diabetes mellitus (T2DM) and it is estimated that this will rise to 5 million by 2025 [2]. PCa and T2DM thus often occur together in the same individual

T2DM increases the risk of cancer-specific death from several solid malignancies, including colorectal and breast cancer [3], but conflicting evidence exists in the case of PCa. The impact of pre-existing T2DM has also been studied in regard to grade and stage of PCa at presentation, with conflicting results [4]. Moreover, there is emerging evidence that the presence of T2DM and other metabolic abnormalities (dyslipidaemia, hypertension, obesity) is associated with a more rapid progression of PCa [5, 6]. This relationship is further complicated by the fact that standard treatment for advanced PCa, androgen deprivation therapy (ADT), has been suggested to increase incidence of T2DM [7],as well as worsen glycaemic control in those with pre-existing T2DM.

To provide a background on the complex association between PCa and T2DM, this review is set out to explore the following areas:

1. Impact of pre-existing T2DM on PCa incidence
2. Impact of pre-existing T2DM on PCa grade and stage
3. Impact of pre-existing T2DM on PCa outcomes and mortality
4. Interplay between T2DM and PCa treatments

## Impact of pre-existing T2DM on PCa incidence

T2DM that increases the risk of some solid malignancies is now well established, although not all the literature is robust [8]. Some studies suggest that people with T2DM are as much as twice as likely to die from cancer than those without [3]. However, the opposite is seen in PCa, with an inverse association reported in several published meta-analyses [8].

Bonovas *et al*. published the first meta-analysis examining T2DM and risk of PCa in 2004. They included 14 studies and concluded that T2DM confers a statistically significant 9% decrease in relative risk of developing PCa [9]. This was followed in 2006 by a meta-analysis conducted by Kasper *et al*. which included 19 studies and reported an inverse relationship of a similar magnitude, relative risk (RR): 0.84 (95%CI: 0.76–0.93) [10]. Following this, Bansal *et al*. published an updated meta-analysis including 45 studies, involving 8.1 million participants and 132,331 PCa cases, which also reports an inverse association with an RR of 0.86 (95% CI 0.80–0.92) [11]. More recently, Gang *et al*. published a further updated meta-analysis reviewing the literature up to and including April 2012. This meta-analysis included 56 studies and also reported an inverse association, RR: 0.88 (95%CI: 0.82–0.93) [12]. Here, we perform a further systematic review of the literature up until June 2017.

### Evidence acquisition

The systematic review was performed in accordance with the Preferred Reporting Items for Systematic Reviews and Meta-Analysis (PRISMA) guidelines [13], with search terms, inclusion and exclusion criteria all defined *a priori*.

### Search strategy

A computerised literature search of Pubmed to identify full text and abstracts published was performed. The search was done with and without MESH terms (diabetes, diabetes mellitus, PCa, prostate neoplasm, incidence, risk). All references of the selected articles were checked, including hand searches.

### Study eligibility

The final articles were chosen based on the following set of inclusion criteria:

- examined association of T2DM with PCa incidence/risk
- case control or cohort study
- English Language
- not included in the prior published meta-analyses described above

Articles were excluded if they:

- examined association of T2DM with PCa mortality
- examined association of T2DM treatments (i.e. drugs) and PCa incidence
- were a review article or meta-analysis

**Figure 1.**
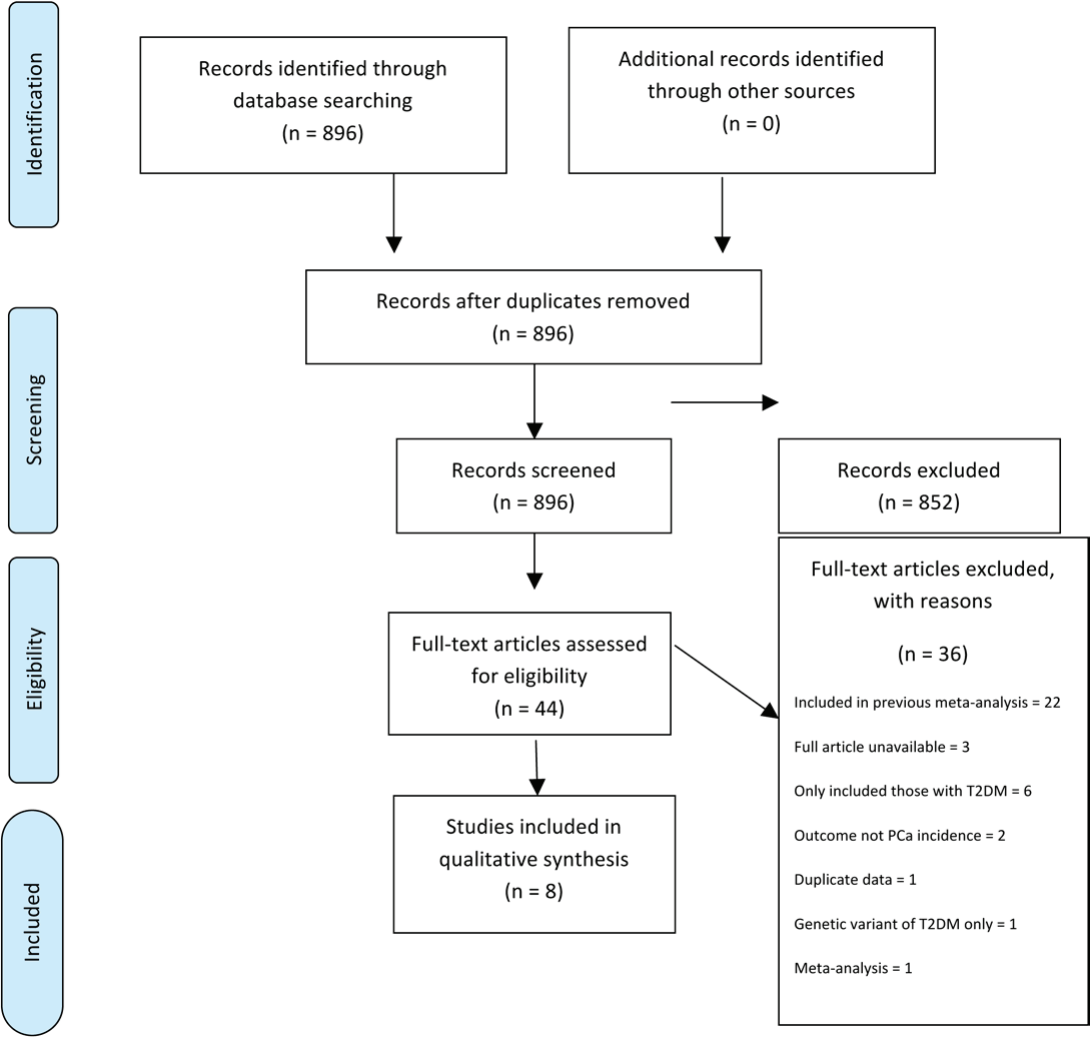
Preferred reporting items for systematic reviews and meta-analysis (PRISMA) flow diagram of article identification, screening, eligibility and inclusion for systematic review on impact of T2DM on PCa incidence.

Initially, titles were reviewed to assess whether they met inclusion criteria. If, after assessing the abstract, there was any doubt regarding whether it met the relevant criteria, it was kept for more thorough, subsequent assessment. The list of potential articles was further shortened by performing detailed evaluations of the methods and results of each remaining paper. Figure 1 provides more detailed information regarding the exclusion process. The strength of each study was assessed using the Strengthening the Reporting of Observational Studies in Epidemiology (STROBE) criteria [14] and is shown in Table 1.

### Data collection

The following details were recorded for each study: author, year of publication, country where study was undertaken, study design, number of patients, population/setting, outcome reported and variables adjusted for in the analysis.

### Evidence synthesis

The literature search identified a total of 896 studies, of which 44 were deemed initially relevant. Using the above inclusion and exclusion criteria, 36 were excluded (Figure 1). The reasons for exclusion were included in previously published meta-analysis (*n* = 22), only T2DM patients included (*n* = 6), full article not available (*n* = 3), outcome not PCa incidence (*n* = 2), meta-analysis (*n* = 1), duplicate data (*n* = 1), genetic variant of T2DM examined only (*n* = 1) (Figure 1). A total of eight studies were included in the systematic review (Table 2).

Of these eight studies, five were cohort studies [15–20] and three case-control studies [21, 22]. Three studies were from European populations, two from the USA, two from Israel and one from Australia.

The studies combined included 2,716,302 subjects. Six reported an inverse association between T2DM and PCa incidence [15–18, 20, 22], one reported no association [21] and in one a positive association was reported [19]. Of the six studies reporting an inverse association, they all reported similar measures of association in the magnitude of a 20% reduction in risk of PCa in those exposed to T2DM as compared to those without T2DM. The one paper which reports no statistically significant association is a case-control study in which cases of T2DM were patients enrolled in the Freemantle Diabetes Study [21]. It is the smallest of the studies, with 1,289 cases and 5,156 controls – which may account for the non- statistical significance of their findings (HR: 0.83, 95%CI: 0.60–1.14) – though the direction and magnitude of the association reported is in line with the other studies. The study which reported a positive association is a retrospective review on 3,162 consecutive men who underwent a prostate biopsy due to either an elevated PSA and/or an abnormal DRE [19]. This design is different from the other studies included here, which were largely based on the general population, not on a selected population attending for a prostate biopsy. This heterogeneity in the design may account for the findings of a 26% increased odds of a positive biopsy in patients with T2DM, compared to those without (OR: 1.26 95%CI: 1.01–1.55).

### Discussion

The biological mechanism underlying the inverse association between T2DM and PCa risk is not elucidated. First, several metabolic alterations occur in people with T2DM which may protect from PCa. The Insulin-IGF-1 theory of carcinogenesis suggests that prolonged hyperinsulinaemia results in reduced insulin binding proteins and therefore increased free IGF-1, which results in cellular changes that can lead to carcinogenesis via increased mitosis and decreased apoptosis [23]. There is both laboratory and epidemiological evidence supporting that raised insulin levels are associated with increased PCa risk [24, 25]. Patients with T2DM, though initially may have raised insulin levels, over time develop hypoinsulinaemia. Hence, patients with T2DM who have lower levels of insulin over time would be protected in terms of PCa risk [26]. Several studies have reported a strengthening of the inverse association between T2DM and PCa risks with the duration of T2DM [27–29], which serves to strengthen this hypothesis. Prolonged hypoinsulinaemia may also result in a reduction of leptin, a hormone involved in energy homeostasis [30], raised levels of which have been associated with PCa risk [31, 32]. However, there are no published studies specifically examining this relationship between insulin and leptin levels in T2DM and risk of PCa.

Another metabolic change which occurs in T2DM is a reduction in testosterone levels which has been shown both in animal models and *in vitro* [33, 34]. PCa is testosterone driven [35]; therefore, a decrease in testosterone is expected to be associated with a decreased risk.

Genetic factors as well as metabolic changes have also been postulated to be involved in the protective effect which T2DM appears to have on PCa risk. The TCF2 gene confers a predisposition to T2DM and has also been shown to have a potential protective effect in PCa. Similarly, other studies have identified different variants in the JAZF1 gene, one associated with T2DM and another associated with PCa [36].

Some cross-sectional studies have shown that men with T2DM have lower PSA levels, compared to those without T2DM [37] and the rate of change over time is also lower [38]. This could result in less screen detected PCa and at least in part account for the difference in the risk seen. This is supported both by studies in which enrolled participants undergo prostate biopsy, which report increased risk of positive biopsies in T2DM [19], and by those which show higher grade PCa detected in those with T2DM [4].

Finally, treatments used for T2DM, including metformin, could be potential confounders in the association between T2DM and PCa risks, which is discussed later in this review.

### Conclusion

The updated systematic review of the literature examining the association between T2DM and PCa risks presented here concurs with the previously published findings of several meta-analyses, indicating that T2DM has a protective effect on PCa risk. However, the underlying biological mechanisms are yet to be elucidated.

## Impact of pre-existing T2DM on PCa grade and stage

PCa severity can be described in terms of its grade and its clinical or TNM stage. Some studies have examined whether pre-existing T2DM is associated with a particular grade or stage of disease. There is considerable overlap with the literature presented above examining T2DM and PCa incidence and that examining particular stage and grade of PCa. In the systematic review of T2DM and PCa incidence above which includes studies published after 2012, of the eight new studies identified, only two presented subgroup analysis on stage and/or grade [15, 18] (Table 3). These are discussed below but another systematic review was not deemed informative, as one including literature up until 2013 has previously been published [4].

### Existing literature

In 2013, Xu *et al*. undertook a meta-analysis of all studies examining the association between T2DM and PCa risks including subgroup analysis by different grade and stage [4]. They included nine studies: five examining stage only, two grade only and two which explored both. They reported findings of an inverse association between T2DM and PCa for both low- and high-grade PCa, defined as Gleason 2–6 and Gleason 7–10 (RR: 0.74, 95%CI: 0.64–0.86 and 0.78, 95%CI: 0.67–0.90). They reported an RR of a similar direction and magnitude for localised and advanced disease (RR: 0.72, 95%CI: 0.68–0.76 and 0.85, 95%CI: 0.75–0.97). This meta-analysis included all studies published up until October 2012.

### Updated literature review

Two new studies were identified in the systematic review described above which considered stage and grade of PCa. A nested case-control study by Fall *et al*. included 44,352 men with PCa in Prostate Cancer data Base Sweden (PCBaSe) Sweden, which is based on the National Prostate Cancer register of Sweden (NPCR) [39]. They showed an inverse association between T2DM and risk of PCa across all risk groups, low, intermediate and high risk/metastatic (OR: 0.71, 95%CI: 0.64–0.80; 0.76, 95%CI: 0.69–0.84; 0.86, 95%CI: 0.80–0.93, respectively). Although they showed a slightly less clear risk pattern for those with high risk and metastatic disease, no significant difference between T2DM and risk category of PCa emerged from this study.

Tsilidis *et al*. included 139,131 men from the European Prospective Investigation into Cancer and Nutrition (EPIC) prospective cohort, 4,531 of whom went on to develop PCa. They reported no statistical evidence for an inverse association between T2DM and PCa risks. There was no evidence that the association differed by stage (p-heterogeneity, 0.19) or grade (p-heterogeneity, 0.48) of the disease, although the numbers were small in some subgroups and the study may have been under powered to detect differences.

### Conclusion

The meta-analysis by Xu *et al*. [4] concluded that the protective effect of T2DM on PCa risk is seen across different disease grades and stages; however, the number of studies available was too small to confirm this finding. The two new studies identified in the systematic review of T2DM and PCa presented above, which considered grade or stage [15, 18], reported similar findings of no significant difference between different stages and grades of PCa. However, sample sizes remain small and no definitive conclusion can be drawn on the impact of T2DM on risk of PCa of different grades and stages. Larger studies are required to address this question in detail.

## Impact of pre-existing T2DM on prostate cancer outcomes and mortality

### Introduction

In 2008, a systematic review and meta-analysis examining all-cause mortality in cancer patients reported worse outcomes in cancer patients who had pre-existing diabetes mellitus (DM) [40]. However, the magnitude of the association varied widely between different types of malignancy. This led to a call for research focusing on individual cancer types. Following this, a body of literature has emerged and three published meta-analyses examined the association between pre-existing DM and PCa specific and all-cause mortality [41–43]. The most recent of which was published in 2016. Much of this literature does not distinguish between type 1 and type 2 diabetes; hence in this section refers to DM, encompassing both types, unless otherwise specified.

### Existing literature

In 2010, Synder *et al*. performed a systematic review of the literature (seven papers), but was only able to include four studies in a meta-analysis. They could only investigate all-cause mortality and reported a pooled HR of 1.57 (95%CI: 1.12–2.20) for DM versus no DM. They concluded that more rigorous research was necessary before firm conclusions could be drawn.

Subsequently, a further meta-analysis by Cai *et al*. [42] was published in 2015, which included 11 cohort studies and looked at outcomes of all-cause mortality, PCa-specific mortality and non-PCa mortality. It reported that DM was positively associated with all three outcomes, with pooled HR of 1.50 (95%CI: 1.25–1.79), 1.26 (95%CI: 1.20–1.33), 1.83 (95%CI: 1.33–2.52), respectively. They concluded that DM was associated with an adverse prognosis in PCa and that clinicians treating patients with both conditions should pay more attention to the dual diagnosis and even consider more aggressive treatment strategies.

The final and most recent meta-analysis by Lee *et al*. [43] included 17 cohort studies which included 274,677 men. The studies were mainly from the USA (eight) and Europe (six), with two from Taiwan and one from Korea. They reported a 29% increase in PCa-specific mortality (95%CI 1.37–2.96), alongside a 37% increase in all-cause mortality (95%CI: 1.29–1.45). They performed a subgroup analysis of three cohort studies which considered T2DM separately from type 1 DM. This analysis showed a twofold increase in all-cause mortality for those with T2DM as compared to those without DM (95%CI:1.37–2.96), and could not exclude a positive association with PCa-specific mortality (RR:1.17, 95%CI: 0.96–1.42).

### Evidence acquisition

As the meta-analysis by Lee was published within the last year, a full systematic review of the literature was not deemed valuable. However, the literature since 2016 was reviewed searching Pubmed using terms (with and without MESH terms): PCa, DM, prognosis and mortality. Only one new paper was identified [44].

### Evidence synthesis

The one new paper identified is by Zaorsky *et al*. It is a retrospective cohort study of 3,217 men with localised PCa undergoing curative radiotherapy. Patients were divided into five groups: 1) No T2DM (*n* = 2,603); 2) T2DM on oral hypoglycaemic agents (OHA) including metformin (*n* = 251); 3) T2DM OHA not including metformin (*n* = 148); 4) T2DM on insulin (*n* = 89); 5) T2DM – diet controlled (*n* = 126). They examined several outcomes including OS, freedom for biochemical failure and cancer-specific survival. They showed an increased overall mortality in those on insulin (HR: 2.06, 95%CI: 1.17–3.63) or with diet-controlled T2DM (HR: 2.01, 95%CI: 1.24–3.26), but only an increase in PCa-specific mortality for those on insulin (HR: 3.91, 95%CI: 1.22–11.46). These findings may suggest that OHAs are potentially protective in PCa, the relationship between metformin and PCa is discussed in detail later in this review. Although interesting, this study contained relatively small numbers in each treatment subgroup making it difficult to interpret the results. It also addressed a slightly different research question than the previously discussed meta-analysis focusing in more detail on the treatment for T2DM, rather than just the presence of absence of T2DM.

### Discussion

All three meta-analyses showed an increased risk of all-cause mortality for patients with DM compared to those without. The magnitude varied from 37% to 57% increased risk. T2DM increases cardiovascular mortality amongst a multitude of other consequences; increased all-cause mortality for those with T2DM is expected. The evidence for PCa-specific mortality is less clear. Both meta-analyses that examined this reported an increased risk in the order of 25–30%; however, when only studies which included those with T2DM and not type 1 DM were analysed this increased risk was not statistically significant, though sample sizes were small for this subgroup analyses. In the age group affected most commonly with PCa, T2DM is more prevalent than type 1 DM, and so it is probably fair to assume that T2DM is largely contributing to the increases in both all-cause and PCa-specific mortality demonstrated.

A further limitation of the existing literature is that some studies failed to adjust for PCa stage or grade, which is an important co variate associated with PCa mortality [45]. Positive associations reported in these studies could be due to failure to adjust for other important co variates, rather than a true effect of DM. Duration and severity of DM are also important co variates which are often not adjusted for. Bensimon *et al*. [46] reported a 23% increased risk of PCa-specific mortality and a 25% increased risk of all-cause mortality in those with T2DM. They also examined the effect of duration of T2DM and found a peak increase in PCa risk between 2 and 8 years. They also performed a sensitivity analysis whereby they excluded those people who developed T2DM during follow up, as this could have diluted the risks seen, however this made no difference.

### Conclusion

The existing literature indicates that T2DM is associated with increased risk of all-cause and probably also PCa-specific mortality. However, limitations in studies hitherto preclude reliable estimates of what the real sizes of the associations are, especially for PCa-specific mortality.

## Interplay between T2DM and PCa treatments

### Androgen deprivation therapy and risk of T2DM

ADT is widely used in the management of PCa. It is the recommended first-line treatment in all men with advanced disease, as well as in men with high-risk disease following radical radiotherapy [47]. Even when PCa progresses to a castrate resistant phenotype, it is recommended that treatment with ADT continue, alongside the addition of further therapies. Given the prolonged clinical course of many men with PCa, they can remain on ADT for many years, making any side effects associated with treatment potentially significant.

Common adverse effects of ADT include fatigue, hot flushes and sexual dysfunction [48]. ADT also increases the risk of cardiovascular disease [49, 50], reduced bone mineral density [51] and several North American cohorts have demonstrated an increased risk of diabetes [7, 52–54]. This led the Food and Drug Administration (FDA) in 2010 to require a risk label on all GnRH agonists for increased risk of diabetes and certain cardiovascular diseases (heart attack, sudden cardiac death and stroke) [55].

**Figure 2.**
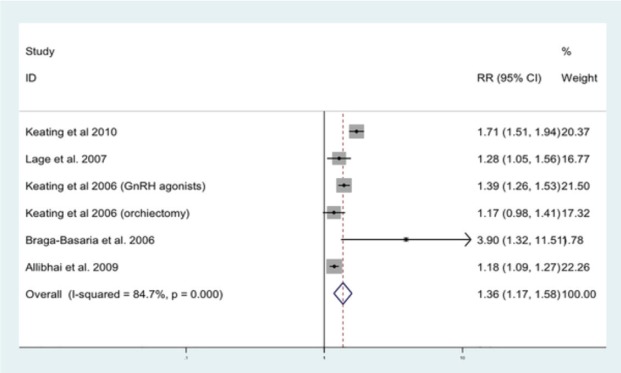
Forrest plot for association between ADT and risk of diabetes [57].

ADT has been shown to induce a metabolic-like syndrome, in which patients have decreased insulin sensitivity and increased body fat [56]. We have previously undertaken a meta-analysis, including nine published studies, to quantify the association between ADT and MetS [57]. The relative risk of MetS for those on ADT compared to PCa men not on ADT was 1.75 (95%CI 1.27–2.41) and for T2DM alone 1.36 (95%CI 1.17–1.58) (Figure 2). Here we performed an up-to-date systematic review of the literature examining the association between T2DM and ADT.

### Evidence acquisition

The systematic review was performed in accordance with the PRISMA guidelines [13] with search terms, inclusion and exclusion criteria all defined *a priori*.

### Search strategy

A computerised literature search of Pubmed to identify full text and abstracts published was performed. The search was done with and without MESH terms (androgen, androgens, deprivation, therapy, therapeutics, diabetes, diabetes mellitus). All references of the selected articles were checked, including hand searches.

### Study eligibility

The final articles were chosen based on the following set of inclusion criteria:

- Original article
- Examined the association of ADT with the risk of developing T2DM
- English language article

Excluded if:

- Review or meta-analysis
- Examined elements of the metabolic syndrome which did not include T2DM (i.e., hyperglycaemia only)

**Figure 3.**
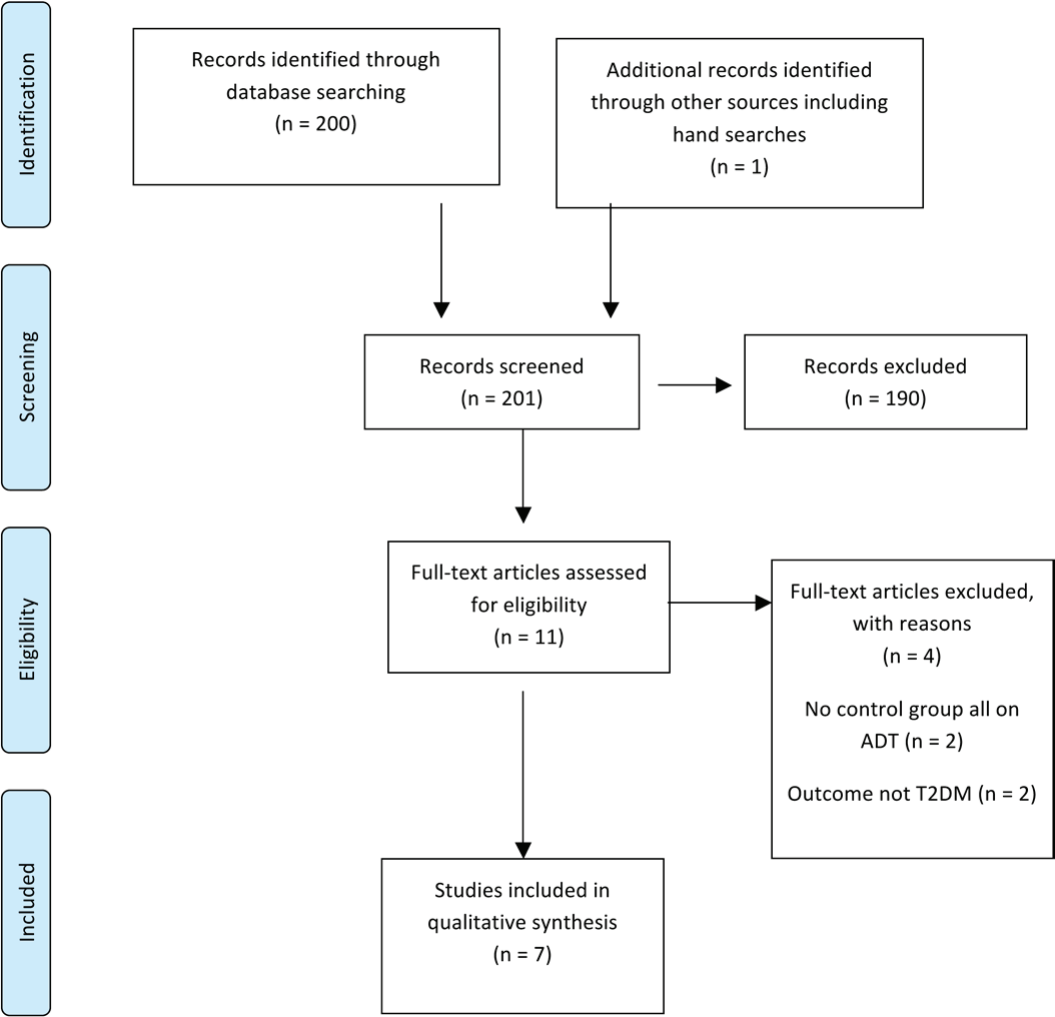
Preferred reporting items for systematic reviews and meta-analysis (PRISMA) flow diagram of article identification, screening, eligibility and inclusion for systematic review on ADT and risk of T2DM.

Initially, titles were reviewed to assess whether they met inclusion criteria. If, after assessing the abstract, there was any doubt regarding whether it met the relevant criteria, it was kept for more thorough, subsequent assessment. The list of potential articles was further shortened by performing detailed evaluations of the methods and results of each remaining paper. Figure 3 provides more detailed information regarding the exclusion process. The strength of each study was assessed using the STROBE criteria [14] and is shown in Table 4.

### Data collection

The following details were recorded for each study: author, year of publication, country where study was undertaken, study design, number of patients, type of ADT, outcome reported and variables adjusted for in the analysis.

### Evidence synthesis

The literature search identified a total of 200 studies of which 10 were deemed as initially relevant and a further one study was identified using hand searches. Using the above inclusion and exclusion criteria, four were excluded (Figure 3). The reasons for exclusion were outcomes not T2DM [2] and no control group not on ADT [2]. Seven studies were included in the systematic review (Table 5).

All seven studies were cohort studies. Five were from North American cohorts [7, 52–54, 58], one European [59] and one Asian [60]. The studies combined include 97,893 men on ADT and 287,312 not on ADT. They all report an increased risk of T2DM in those men receiving ADT compared to those men that are not, particularly in those receiving GNRH agonists. The magnitude of this risk varies from a 16% increase reported by Alibhai *et al*. [52] to 61% [58, 59]. The one outlier is the study by *Teoh et al*. [60] which reports a much higher increased risk, HR: 3.34 (95%CI: 1.19–9.39). However, this is a much smaller study in comparison with just a few hundred patients compared to the other cohorts which include several thousand patients. This is reflected in the wide confidence intervals surrounding the HR that they present.

Most of the studies in this systematic review included data only on GnRH agonists and orchidectomy or combined all forms of ADT. Only two studies examine anti-androgens (AA) separately [54, 59] and both report no increased risk with those receiving AA alone.

The impact of the duration for which ADT was received and T2DM risk had been examined in two studies [7, 52] but with a relatively short exposure time (25 months). We have previously studied in detail the impact of duration of ADT on risk of T2DM, with exposure times of up to greater than 10 years [59]. This paper showed that the peak risk of T2DM in men receiving GnRH agonists/orchiectomy was within the first three years of exposure [i.e., 1–1.5 years; HR: 1.61 (95%CI: 1.36–1.91)], before the risk off with continued exposure. Thereby showing that the duration of ADT is also important with regards the risk of T2DM.

### Discussion

There is good concordance between all studies examining the risk of T2DM with ADT, with all showing an increased risk. The large North American cohort studies, which led to the FDA requiring a risk label on all GnRH agonists for increased risk of T2DM back in 2010, have since been corroborated by further studies in both European [59] and Asian populations [60]. Additionally, the literature demonstrates that both the type and duration of the ADT are important in the risk of T2DM and should be considered by physicians prescribing ADT. The observed temporal changes in risk fit with the physiological and metabolic changes previously described for GnRH agonist treatment [61]. These changes included increased fat mass, reduced lean body mass and increased insulin levels, which all have been demonstrated to occur within three months of commencing ADT [61–63]. Lee *et al*. measured lean body mass and fat mass in 65 men with PCa on GnRH agonists over a 12-month period. Those with longer prior exposure to treatment with GnRH agonists had less fat accumulation and less loss of lean body mass over the 12-month period [62]. Similarly, GnRH agonists decrease sensitivity to insulin within three months of ADT start [64]. Thus, the adverse metabolic effects of GnRH agonists occur within months of initiation; the consequences of these changes (i.e., developing T2DM) do not peak until several years later.

### Conclusion

The literature consistently reports that ADT increases the risk of T2DM and, furthermore, that the type and duration of that ADT is important in determining that risk.

## Impact of T2DM treatments on PCa: metformin and PCa

### Introduction

Metformin (1,1-dimethylbiguanide hydrochloride) is a biguanide class of OHA and is commonly used for the treatment of T2DM. Metformin inhibits gluconeogenesis and reduces circulating levels of insulin [65]. It is also thought to play a role in lowering triglyceride and LDL cholesterol levels [66]. In addition to its anti-diabetic effect, metformin has also been associated with a reduced risk of various cancers including PCa [67–69]. The literature has reported inconsistent results and several meta-analyses have been undertaken in attempt to clarify results [70–73]. These are summarised below.

### Existing literature: metformin and PCa risk

In 2015, Deng *et al*. [72] reported a decrease in risk of PCa with metformin in a meta-analysis which included seven studies (RR: 0.88; 95%CI: 0.78–0.99). Conversely in the same year, Wu *et al*. [70] included six cohort and four case-control studies in a meta-analyses and reported no association with PCa risk (RR: 0.92; 95%CI: 0.84–1.02). However, when only cohort studies were considered, a small but statistically significant reduction in risk was reported (RR: 0.92; 95%CI: 0.87–0.96). Similarly in a larger meta-analysis by Gandini *et al*. in 2014 [69], metformin treatment and PCa risk did not show any association in 12 studies (SRR: 1.06; 95%CI: 0.80–1.41), though a small but statistically significant association was seen when considering just the six prospective studies (SRR: 0.93; 95%CI: 0.89–0.97).

### Existing literature: metformin and PCa mortality and outcomes

The meta-analysis by Deng *et al*. [72] described above also examined how metformin exposure was associated with all-cause mortality (three studies) and biochemical recurrence (BCR) of PCa (four studies). They reported that metformin exposure was not associated with either all-cause mortality (RR: 1.07; 95%CI: 0.86–1.32) or BCR (RR: 0.90; 95%CI: 0.75–1.09). Also in 2015, Raval *et al*. (73) published a systematic review and meta-analysis of the impact of metformin exposure on clinical outcomes in PCa. They also report no association with all-cause mortality and PCa specific mortality, but reported a marginal association with reduced risk of BCR in five studies (HR: 0.59; 95%CI: 0.67–1.01). In a further meta-analysis by Stopsack *et al*. [71] in 2016, including nine retrospective cohort studies of 9,186 patients, once again no overall association with PCa-specific mortality was seen, but metformin exposure was associated with improved OS in these studies (HR: 0.88; 95%CI: 0.86–0.90) and with a decreased risk of BCR (HR: 0.79; 95%CI: 0.63–1.00).

### Evidence acquisition

As several full meta-analysis and systematic reviews were published in 2015/2016, a full systematic review of the literature was not deemed valuable. However, the literature since 2015 was reviewed searching PubMed using terms (with and without MESH terms): metformin, PCa, risk, mortality and outcomes. Two additional studies were identified [74, 75].

### Evidence synthesis

The two new studies identified are summarised in Table 6. Both were large Scandinavian cohort studies. Haggstrom *et al*. [74] reported that men with more than one-year duration of T2DM had a reduced PCa risk, but that those receiving metformin specifically did not (HR: 0.96; 95%CI: 0.77–1.19). Conversely, the Finnish study by Haring *et al*. [75] reported that men using antidiabetic drugs had lowered overall PCa risk (HR: 0.85; 95%CI: 0.79–0.92) and among antidiabetic drug users, metformin decreased overall PCa risk (HR: 0.81; 95%CI: 0.69–0.95) in a dose-dependent manner.

### Discussion

Despite many studies examining the impact of metformin on PCa risk and outcomes they continue to offer conflicting results. This may be explained by the wide heterogeneity in the design and quality of these studies. Similarly, the meta-analysis, which has been performed to provide clarity, has also provided conflicting results. In their meta-analysis, Stopsack *et al*. [71] attempted to take into account the differing designs and quality of the literature. In their primary analysis, they only included studies with a clear risk window and in a secondary analysis examined those studies with potential immortal time bias. They showed that an otherwise modest association with reduced PCa risk was magnified in the studies with potential immortal time bias (HR: 0.52; 95%CI: 0.41–0.65). This highlights the need to take into account the quality of studies when performing meta-analysis. Two well-designed and well-powered Canadian studies by Margel *et al*. [68, 76] reported that an increased cumulative duration of metformin exposure after PC diagnosis was associated with decreases in both all-cause and PC-specific mortality among diabetic men, but was not associated with PCa incidence.

Several biological mechanisms underlying these potential associations have been proposed [77]. One hypothesis is that its anti-neoplastic effect may be via an indirect effect of insulin lowering, which in turn leads to a reduction in IGF-1 levels. Both elevated insulin and IGF-1 levels are known to play a role in PCa development and progression [78]. However, a host of direct molecular mechanisms has also been suggested. Many of these actions are mediated via 5’-AMP- activated protein kinase (AMPK), which is activated under conditions of metabolic stress that leads to intracellular adenosine triphosphate (ATP) being depleted and AMP increasing. Once activated, AMPK inhibits the mammalian target of rapamycin (mTOR) and other protein synthesis. These direct effects can lead to reduced cell proliferation [79] and hence exert an anti-cancer effect. Metformin is a potent activator of AMPK via inhibition of complex I of the respiratory chain, which results in increased AMP [80].

### Conclusion

The current epidemiological evidence shows neither a conclusive decrease in PCa risk or improvement in PCa or all-cause mortality with metformin. Further rigorous, well-designed and powered studies are needed to clarify these potential associations.

## Impact of PCa on T2DM control and treatments

### Introduction

The relationship between PCa and T2DM has been extensively studied with respect to the effects of T2DM on PCa risk and progression, as described above. However, conversely the impact of a PCa diagnosis on the treatment of T2DM has received less attention. In this final section, this review considers the impact of PCa on T2DM control and treatments. In particular, PCa treatments including ADT and corticosteroids given alongside chemotherapy may have an impact on the management of pre-existing T2DM. However, there is little literature in this area. Below we have performed a systematic review of what has been published to date on this subject.

### Evidence acquisition

The systematic review was performed in accordance with the PRISMA guidelines [13] with search terms, inclusion and exclusion criteria all defined *a priori*.

### Search strategy

A computerised literature search of Pubmed to identify full text and abstracts published was performed. The search was done with and without MESH terms (PCa, diabetes control). All references of the selected articles were checked, including hand searches.

### Study eligibility

The final articles were chosen based on the following set of inclusion criteria:

- Original epidemiological study
- Examined the impact of PCa diagnosis or treatment on T2DM control or treatment
- English language article

The studies were excluded if:

- Review or meta-analysis

**Figure 4.**
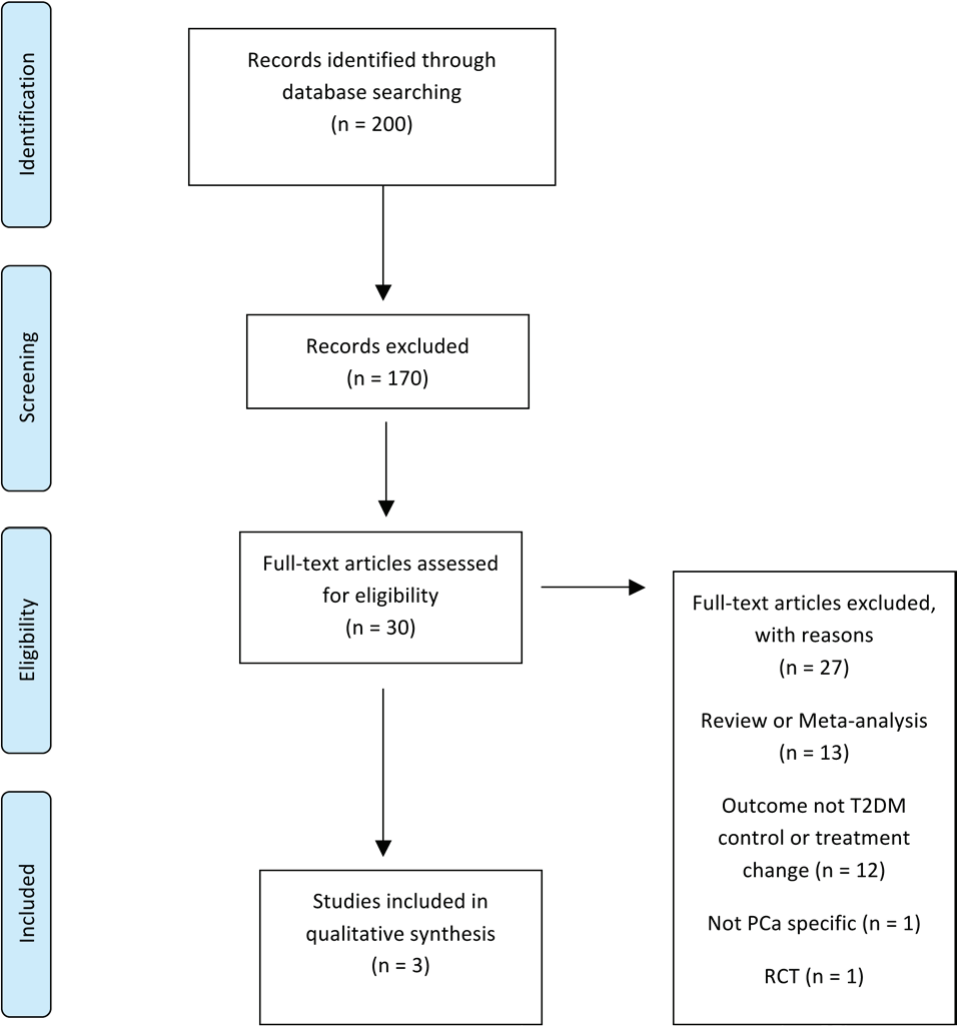
Preferred reporting items for systematic reviews and meta-analysis (PRISMA) flow diagram of article identification, screening, eligibility and inclusion for systematic review on impact of PCa on T2DM control and treatment.

Initially, titles were reviewed to assess whether they met inclusion criteria. If, after assessing the abstract, there was any doubt regarding whether it met the relevant criteria, it was kept for more thorough, subsequent assessment. The list of potential articles was further shortened by performing detailed evaluations of the methods and results of each remaining paper. Figure 4 shows more detailed information regarding the exclusion process. The strength of each study was assessed using the STROBE criteria [14] and is shown in Table 7.

### Data collection

The following details were recorded for each study: author, year of publication, country where study was undertaken, study design, number of patients, main outcome and main findings.

### Evidence synthesis

The literature search identified a total of 200 studies of which 30 were deemed as initially relevant. Using the above inclusion and exclusion criteria, 27 were excluded (Figure 4). The reasons for exclusion were review or meta-analysis (*n* = 13), outcome not T2DM controls or treatment change (*n* = 12), not PCa specific (*n* = 1), RCT (*n* = 1). Three studies were included in the systematic review (Table 8).

All three studies identified were North American cohort studies [81–83]. By far the largest of these studies was by Keating *et al*. [81] which included 2,237 pairs of propensity matched men with PCa and T2DM who did or did not receive ADT. They calculated mean HbA1c at baseline for both the ADT and No ADT groups and then examined the difference in difference at baseline, one and two years between the groups. They reported that HbA1c increased at one year for men treated with ADT to 7.38 and decreased among men not treated with ADT to 7.14, for a difference in differences of + 0.24 (*P* = 0.008). Results were similar at two years (*P* = 0.03). They also performed Cox proportional hazards regression model in the propensity matched data to assess if ADT was associated with initiating or adding a new class of anti-diabetes drug. They reported an increased risk of initiating an additional anti-diabetic medication in those men on ADT (HR: 1.20; 95%CI: 1.09–1.32), despite the rise in HbA1c seen in those receiving ADT.

Derweesh *et al*. [82] also examined glycaemic control in men with pre-existing T2DM starting on ADT. Glycaemic control was defined by comparing mean fasting blood glucose and HbA1c levels before and after treatment. At least three separate values for FBG and HbA1c were averaged to obtain mean values before and after ADT for comparison. Subsets were then analysed to determine the percentage of patients with a ≥ 10% rise in mean FBG or mean HbA1c after starting ADT. They reported an increase of ≥ 10% in serum HbA1c in 15 patients (19.5%) and an increase of ≥ 10% in FBG in 22 patients (28.6%). However, there was no comparison group in this study.

The final study identified in this systematic review is a descriptive cohort study of 30 patients with T2DM and genitourinary cancer, 26 of whom had PCa, who were receiving corticosteroids alongside their chemotherapy [83]. They examined changes in T2DM treatment and hospitalisations due to hyperglycaemia and reported that 40% of patients required a change in their T2DM management (*n* = 4) and 20% (*n* = 2) required hospitalisations for hyperglycaemia.

### Discussion

This systematic review has highlighted a gap in the existing literature examining the impact of PCa and its treatments on the control and management of pre-existing T2DM. The studies published and described above all suggest that PCa treatments, including ADT and corticosteroids, do impact the management of pre-existing T2DM. However, the existing studies are very limited.

### Conclusion

There is a need for further original research into this area, as there is little research evidence available.

## Summary of findings

This review began with a systematic review of the impact of pre-existing T2DM on PCa incidence and corroborates previously published findings, indicating that T2DM has a protective effect on PCa risk. Several potential biological mechanisms and possible biases to explain this inverse association were discussed. Secondly, the impact of pre-existing T2DM on stage and grade was explored. Some studies have suggested that the inverse association is seen only in low-risk cancers and that those with T2DM are actually more likely to have higher grade and stage PCa. However, this is not consistent with existing meta-analysis and at present no conclusion can be drawn on the impact of T2DM on the PCa risk of different grades and stages. The existing literature does, however, show that T2DM is consistently associated with increased risk of all-cause and PCa-specific mortality. The relationship between T2DM and PCa is further complicated by the interaction between the two conditions and their treatments. The relationship between ADT and T2DM was examined and there is good concordance between all studies, with all showing an increased risk of T2DM. The epidemiological evidence examining the relationship between metformin exposure and PCa, however, is less convincing. It shows neither a conclusive decrease in the PCa risk or improvement in PCa or all-cause mortality with metformin. Finally, we have highlighted a gap in the existing literature examining the impact of PCa and its treatments on the control and management of pre-existing T2DM. Further research is needed in this area.

## Figures and Tables

**Table 1.** Strengthening the Reporting of Observational Studies in Epidemiology (STROBE) checklist for observational studies included in the systematic review on impact of T2DM on PCa incidence.

	Item No.	Recommendation	Dankner 2006 [20]	Tsilidis 2015 [15]	Lai 2013 [16]	Lawrence 2013 [17]	Fall 2013 [18]	Magliano 2012 [21]	Attner 2012 [22]	Moses 2012 [19]
**Title and abstract**	1	(a) Indicate the study’s design with a commonly used term in the title or the abstract	☒	☐	☒	☐	☒	☒	☒	☒
		(b) Provide in the abstract an informative and balanced summary of what was done and what was found	☒	☒	☒	☒	☒	☒	☒	☒
**Introduction**										
Background/rationale	2	Explain the scientific background and rationale for the investigation being reported	☐	☒	☐	☒	☒	☒	☒	☒
Objectives	3	State specific objectives, including any pre specified hypotheses	☒	☒	☒	☒	☒	☒	☒	☒
**Methods**										
Study design	4	Present key elements of study design early in the paper	☒	☒	☒	☒	☒	☐	☒	☒
Setting	5	Describe the setting, locations, and relevant dates, including periods of recruitment, exposure, follow-up, and data collection	☒	☒	☒	☒	☒	☒	☒	☒
Participants	6	(*a*) *Cohort study*—Give the eligibility criteria, and the sources and methods of selection of participants. Describe methods of follow-up	☒	☒	☒	☒	*n/a*	*n/a*	*n/a*	☒
		*Case-control study*—Give the eligibility criteria, and the sources and methods of case ascertainment and control selection. Give the rationale for the choice of cases and controls	*n/a*	*n/a*	*n/a*	*n/a*	☒	☒	☒	*n/a*
		(*b*) *Cohort study*—For matched studies, give matching criteria and number of exposed and unexposed	☒	☒	☒	☒	*n/a*	*n/a*	*n/a*	☒
		*Case-control study*—For matched studies, give matching criteria and the number of controls per case	*n/a*	*n/a*	*n/a*	*n/a*	☒	☒	☒	*n/a*
Variables	7	Clearly define all outcomes, exposures, predictors, potential confounders, and effect modifiers. Give diagnostic criteria, if applicable	☒	☒	☐	☒	☒	☒	☐	☒
Data sources/measurement	8*	For each variable of interest, give sources of data and details of methods of assessment (measurement). Describe comparability of assessment methods if there is more than one group	☒	☒	☐	☒	☒	☒	☐	☒
Bias	9	Describe any efforts to address potential sources of bias	☐	☒	☒	☒	☒	☒	☐	☒
Study size	10	Explain how the study size was arrived at	☒	☐	☐	☐	☐	☐	☐	☐
Quantitative variables	11	Explain how quantitative variables were handled in the analyses. If applicable, describe which groupings were chosen and why	*n/a*	☒	*n/a*	☒	☒	☒	*n/a*	☒
Statistical methods	12	(a) Describe all statistical methods, including those used to control for confounding	☒	☒	☒	☒	☒	☒	☒	☒
		(b) Describe any methods used to examine subgroups and interactions	☒	☒	☒	☒	☒	☒	☒	☒
		(c) Explain how missing data were addressed	☐	☒	☐	☐	☐	☐	☐	☒
		(*d*) *Cohort stud*y—If applicable, explain how loss to follow-up was addressed	☐	☒	☒	☐	*n/a*	*n/a*	*n/a*	☒
		*Case-control study*—If applicable, explain how matching of cases and controls was addressed	*n/a*	*n/a*	*n/a*	*n/a*	☒	☒	☒	*n/a*
		(e) Describe any sensitivity analyses	☒	☒	☒	☒	☒	☒	☒	☒
**Results**										
Participants	13*	(a) Report numbers of individuals at each stage of study—e.g., numbers potentially eligible, examined for eligibility, confirmed eligible, included in the study, completing follow-up and analysed	☒	☒	☐	☒	☒	☐	☐	☒
		(b) Give reasons for non-participation at each stage	☐	☐	☐	☐	☐	☐	☐	☐
		(c) Consider the use of a flow diagram	☒	☐	☐	☐	☐	☐	☐	☐
Descriptive data	14*	(a) Give characteristics of study participants (e.g., demographic, clinical, social) and information on exposures and potential confounders	☒	☒	☐	☒	☒	☒	☐	☒
		(b) Indicate number of participants with missing data for each variable of interest	☒	☒	☐	☐	☐	☐	☐	☒
		(c) *Cohort study*—Summarise follow-up time (e.g., average and total amount)	☒	☒	☐	☒	*n/a*	*n/a*	*n/a*	*n/a*
Outcome data	15*	*Cohort study*—Report numbers of outcome events or summary measures over time	☒	☒	☐	☒	*n/a*	*n/a*	*n/a*	☐
		*Case-control study—*Report numbers in each exposure category, or summary measures of exposure	*n/a*	*n/a*	*n/a*	*n/a*	☒	☒	☒	*n/a*
Main results	16	(*a*) Give unadjusted estimates and, if applicable, confounder-adjusted estimates and their precision (e.g., 95% confidence interval). Make clear which confounders were adjusted for and why they were included	☐	☒	☐	☐	☐	☒	☒	☒
		(*b*) Report category boundaries when continuous variables were categorised	*n/a*	☒	*n/a*	☒	☒	☒	*n/a*	☒
		(c) If relevant, consider translating estimates of relative risk into absolute risk for a meaningful time period	☐	☐	☐	☐	☐	☐	☐	☐
Other analyses	17	Report other analyses done—e.g., analyses of subgroups and interactions, and sensitivity analyses	☒	☒	☒	☐	☒	☒	☐	☒
**Discussion**										
Key results	18	Summarise key results with reference to study objectives	☒	☒	☒	☒	☒	☒	☒	☒
Limitations	19	Discuss limitations of the study, taking into account sources of potential bias or imprecision. Discuss both direction and magnitude of any potential bias	☒	☐	☒	☒	☒	☒	☒	☒
Interpretation	20	Give a cautious overall interpretation of results considering objectives, limitations, multiplicity of analyses, results from similar studies, and other relevant evidence	☒	☒	☒	☒	☒	☒	☒	☒
Generalisability	21	Discuss the generalisability (external validity) of the study results	☒	☐	☐	☒	☐	☒	☐	☐
**Other information**										
Funding	22	Give the source of funding and the role of the funders for the present study and, if applicable, for the original study on which the present article is based	☒	☒	☒	☒	☒	☒	☒	☐

**Table 2.** Characteristics of the eight studies included in the systematic review on impact of T2DM on PCa incidence.

Author, year, country	Study design	No. of patients	Population/setting	Outcome reported	Adjusted for
Dankner R, 2016, Israel [20]	Retrospective cohort	2,186,196	Men aged 21–89 covered by a large healthcare provider	T2DM inversely associated with PCa HR: 0.80; 95%CI: 0.76–0.85	Age, ethnicity, socioeconomic status
Tsilidis K, 2015, Europe [15]	Prospective cohort	139,131	Men aged 35–70 from general population	T2DM inversely associated with PCa HR: 0.74; 95%CI: 0.63–0.86	Education, smoking, BMI, waist circumference, physical activity
Lai G, 2013, USA [16]	Prospective cohort	295,276	Men aged 50–71 in six US states, general population	T2DM inversely associated with PCa HR: 0.74; 95%CI: 0.70–0.78	Age, BMI, race, education, marital status, education, family history cancer, diet, smoking
Lawrence YR, 2013, Israel [17]	Prospective cohort within RCT	11,541	Men aged 36–74 with coronary heart disease enrolled in a secondary prevention trial	T2DM inversely associated with PCa HR: 0.54; 95%CI: 0.40–0.73	Fasting glucose, triglycerides, HDL, blood pressure, insulin, tobacco, metformin
Fall, 2013, Sweden [18]	Nested case control	44,352	Men from PCBaSe Sweden	T2DM inversely associated with PCa OR: 0.80; 95%CI: 0.76–0.85	Socioeconomic status, marital status, comorbidity, age at PCa diagnosis, prevalence of DM in county
Magliano DJ, 2012, Australia [21]	Case control	1,289 cases 5,156 controls	Cases from Fremantle Diabetes Cohort Study and controls from general population	No significant association reported HR: 0.83; 95%CI: 0.60–1.14	Age, sex, post code matched controls
Attner B, 2012, Sweden [22]	Case control	3,545 cases 26,654 controls	Cases from Cancer register Southern Sweden, controls from general population	T2DM inversely associated with PCa RR: 0.81; 95%CI: 0.72–0.93	Age, sex, county matched controls
Moses KA, 2012, USA [19]	Retrospective cohort	3,162	Men referred for a prostate biopsy because of abnormal DRE and/or abnormal PSA	T2DM associated with increased odds of positive biopsy OR: 1.26; 95%CI: 1.01–1.55	Age, race, BMI, prostate volume, family history, PSA, DRE, interaction PSA and DRE

**Table 3.** Overview of the eight papers included in the systematic review on T2DM and PCa incidence, by subgroup analysis including PCa stage and grade.

Author, year, country	PCa stage	PCa grade
Dankner R, 2016, Israel [20]	No	No
Tsilidis K, 2015, Europe [15]	Yes	Yes
Lai G, 2013, USA [16]	No	No
Lawrence YR, 2013, Israel [17]	No	No
Fall 2013, Sweden [18]	Yes	Yes
Magliano DJ, 2012, Australia [21]	No	No
Attner B, 2012, Sweden [22]	No	No
Moses KA, 2012, USA [19]	No	No

**Table 4.** Strengthening the Reporting of Observational Studies in Epidemiology (STROBE) checklist for observational studies included in the systematic review of ADT and T2DM.

	Item No.	Recommendation	Keating (2006) [7]	Lage (2007) [53]	Alibhai (2009) [52]	Keating (2010) [54]	Teoh (2015) [60]	Tsai (2015) [58]	Crawley (2016) [59]
**Title and abstract**	1	(a) Indicate the study’s design with a commonly used term in the title or the abstract	☒	☒	☒	☒	☐	☒	☒
		(b) Provide in the abstract an informative and balanced summary of what was done and what was found	☒	☒	☒	☒	☒	☒	☒
**Introduction**									
Background/rationale	2	Explain the scientific background and rationale for the investigation being reported	☒	☒	☒	☒	☒	☒	☒
Objectives	3	State-specific objectives, including any pre-specified hypotheses	☒	☒	☒	☒	☒	☒	☒
**Methods**									
Study design	4	Present key elements of study design early in the paper	☒	☒	☒	☒	☒	☒	☒
Setting	5	Describe the setting, locations, and relevant dates, including periods of recruitment, exposure, follow-up, and data collection	☒	☒	☒	☒	☐	☒	☒
Participants	6	(a) *Cohort study*—Give the eligibility criteria, and the sources and methods of selection of participants. Describe methods of follow-up	☒	☒	☒	☒	☐	☒	☒
		*Case-control study*—Give the eligibility criteria, and the sources and methods of case ascertainment and control selection. Give the rationale for the choice of cases and controls	*n/a*	*n/a*	*n/a*	*n/a*	*n/a*	*n/a*	*n/a*
		(b) *Cohort study*—For matched studies, give matching criteria and number of exposed and unexposed	☒	☒	☒	☒	☐	☒	☒
		*Case-control study*—For matched studies, give matching criteria and the number of controls per case	*n/a*	*n/a*	*n/a*	*n/a*	*n/a*	*n/a*	*n/a*
Variables	7	Clearly define all outcomes, exposures, predictors, potential confounders, and effect modifiers. Give diagnostic criteria, if applicable	☒	☒	☒	☒	☐	☒	☒
Data sources/measurement	8*	For each variable of interest, give sources of data and details of methods of assessment (measurement). Describe comparability of assessment methods if there is more than one group	☒	☒	☒	☒	☒	☒	☒
Bias	9	Describe any efforts to address potential sources of bias	☒	☒	☒	☒	☒	☒	☒
Study size	10	Explain how the study size was arrived at	☐	☒	☐	☐	☐	☒	☐
Quantitative variables	11	Explain how quantitative variables were handled in the analyses. If applicable, describe which groupings were chosen and why	*n/a*	*n/a*	*n/a*	☒	☒	*n/a*	*n/a*
Statistical methods	12	(*a*) Describe all statistical methods, including those used to control for confounding	☒	☒	☒	☒	☒	☒	☒
		(*b*) Describe any methods used to examine subgroups and interactions	☒	☐	☒	☒	☐	☒	☒
		(*c*) Explain how missing data were addressed	☐	☐	☐	☒	☐	☐	☐
		(*d*) *Cohort study*—If applicable, explain how loss to follow-up was addressed	☐	☐	☒	☐	☐	☒	☐
		*Case-control study*—If applicable, explain how matching of cases and controls was addressed	*n/a*	*n/a*	*n/a*	*n/a*	*n/a*	*n/a*	*n/a*
		(*e*) Describe any sensitivity analyses	☒	☒	☒	☒	☐	☒	☒
**Results**									
Participants	13*	(a) Report numbers of individuals at each stage of study—e.g., numbers potentially eligible, examined for eligibility, confirmed eligible, included in the study, completing follow-up, and analysed	☒	☒	☒	☒	☐	☒	☒
		(b) Give reasons for non-participation at each stage	☐	☐	☒	☐	☐	☐	☐
		(c) Consider the use of a flow diagram	☐	☐	☒	☐	☐	☒	☐
Descriptive data	14*	(a) Give characteristics of study participants (e.g., demographic, clinical, social) and information on exposures and potential confounders	☒	☒	☒	☒	☒	☒	☒
		(b) Indicate number of participants with missing data for each variable of interest	☒	☐	☐	☒	☐	☐	☒
		(c) *Cohort study*—Summarise follow-up time (e.g., average and total amount)	☒	☐	☒	☒	☒	☒	☒
Outcome data	15*	*Cohort study*—Report numbers of outcome events or summary measures over time	☒	☐	☐	☒	☒	☒	☒
		*Case-control study—*Report numbers in each exposure category, or summary measures of exposure	*n/a*	*n/a*	*n/a*	*n/a*	*n/a*	*n/a*	*n/a*
Main results	16	(*a*) Give unadjusted estimates and, if applicable, confounder-adjusted estimates and their precision (e.g., 95% confidence interval). Make clear which confounders were adjusted for and why they were included	☐	☐	☐	☐	☐	☐	☒
		(*b*) Report category boundaries when continuous variables were categorised	*n/a*	*n/a*	*n/a*	*n/a*	*n/a*	*n/a*	*n/a*
		(*c*) If relevant, consider translating estimates of relative risk into absolute risk for a meaningful time period	☒	☐	☐	☒	☐	☒	☒
Other analyses	17	Report other analyses done—e.g., analyses of subgroups and interactions, and sensitivity analyses	☒	☒	☒	☒	☐	☒	☒
**Discussion**									
Key results	18	Summarise key results with reference to study objectives	☒	☒	☒	☒	☒	☒	☒
Limitations	19	Discuss limitations of the study, taking into account sources of potential bias or imprecision. Discuss both direction and magnitude of any potential bias	☒	☒	☒	☒	☒	☒	☒
Interpretation	20	Give a cautious overall interpretation of results considering objectives, limitations, multiplicity of analyses, results from similar studies, and other relevant evidence	☒	☒	☒	☒	☒	☒	☒
Generalisability	21	Discuss the generalisability (external validity) of the study results	☒	☒	☒	☒	☐	☒	☐
**Other information**									
Funding	22	Give the source of funding and the role of the funders for the present study and, if applicable, for the original study on which the present article is based	☒	☒	☒	☒	☒	☒	☒

**Table 5.** Characteristics of the eight Studies included in the systematic review on ADT and T2DM.

Author, year, country	Study design	No. of patients	ADT type	Main findings	Adjusted for
Crawley D, 2016, Sweden [59]	Prospective cohort	34031 ADT vs. 167,205 No ADT	AA, GNRH agonists, Orch	Increased risk GnRH agonists vs. PCa free men HR 1.61 (95%CI: 1.36 – 1.91) No increased risk AA HR 0.74 (95%CI: 0.65 –0.84).	CCI, PCa risk category, education status
Tsai HT, 2015, USA [58]	Retrospective cohort	2648 ADT vs. 9543 No ADT	GNRH agonist +/- AA	Increased risk with ADT vs. No ADT HR 1.61 (95%CI 1.38–1.88)	Age, race, ethnicity, year of diagnosis, cancer sequence, health plan
Teoh JY, 2015, Asia [60]	Retrospective Cohort	219 ADT vs. 169 No ADT	?not in abstract?	Increased risk GnRH agonist HR 3.34 (95%CI 1.19–9.39)Orchiectomy HR 6.49 (95%CI 1.48–28.55) vs. No ADT	Age, T Stage, Gleason score, hypertension, dyslipidaemia, ischaemic heart disease, stroke, follow up time, type of ADT, duration of ADT
Keating NL, 2010, USA [54]	Retrospective cohort	14,597 ADT vs. 37,443 No ADT	AA, GNRH agonists, CAB, Orch	Increased risk with GnRH agonist vs No ADT 1.28 (95%CI 1.19–1.38) No increased risk with AA HR 1.02 (95%CI 0.72–1.45)	Age, race, ethnicity, year of diagnosis, marital status, socioeconomic status, Pca stage and grade, primary treatment, PSA at diagnosis, co morbidities, statin use, finasteride use
Alibhai SM, 2009, Canada [52]	Retrospective cohort	19, 076 ADT vs. 19076 No ADT	LHRH agonists, AA, CAB	Increased risk HR 1.16 (95%CI: 1.11–1.21)	Income and rurality
Lage MJ, 2007, USA [53]	Retrospective claims cohort	1231 ADT vs. 7250 No ADT	Any ADT	Increased risk with ADT HR 1.36 (95%CI 1.07–1.74)	Demographic factors, co morbid conditions, prior statin use
Keating NL, 2006, USA [7]	Retrospective Cohort	26,570 ADT vs. 46,626 No ADT	GNRH agonist, Orch	GnRH agonists HR 1.44 (95%CI 1.34–1.55) vs. No ADT Orch HR 1.34 (95%CI 1.20–1.50) vs. No ADT	Age, race, Hispanic ethnicity, marital status, residence, SEER region, income and education, tumour grade, comorbidity score, year of diagnosis, primary surgical therapy, prevalent coronary heart disease

**Table 6.** Two additional studies identified in systematic review of metformin and PCa risk and outcomes.

Author, year, country	Study design	No of patients	Main findings	Adjusted for
Haring, Finland, 2017 [75]	Cohort	78,615	Metformin decreased PCa incidence in a dose dependent manner (HR 0.81, 95%CI 0.69–0.95)	Age, trial arm, medications
Haggstrom, Sweden, 2017 [74]	Cohort	612,846	Metformin did not decrease PCa incidence (HR 0.96 95%CI 0.77–1.19)	Age, education, CCI, county

**Table 7.** Strengthening the Reporting of Observational Studies in Epidemiology (STROBE) checklist for observational studies included in the systematic review of ADT and T2DM.

	Item No.	Recommendation	Rowbottom (2015) [83]	Keating (2014) [81]	Derweesh (2007) [82]
**Title and abstract**	1	(*a*) Indicate the study’s design with a commonly used term in the title or the abstract	☒	☒	☒
		(*b*) Provide in the abstract an informative and balanced summary of what was done and what was found	☒	☒	☒
**Introduction**					
Background/rationale	2	Explain the scientific background and rationale for the investigation being reported	☒	☒	☒
Objectives	3	State specific objectives, including any pre specified hypotheses	☒	☒	☒
**Methods**					
Study design	4	Present key elements of study design early in the paper	☒	☒	☒
Setting	5	Describe the setting, locations, and relevant dates, including periods of recruitment, exposure, follow-up, and data collection	☒	☒	☒
Participants	6	(a) *Cohort study*—Give the eligibility criteria, and the sources and methods of selection of participants. Describe methods of follow-up	☐	☒	☒
		*Case-control study*—Give the eligibility criteria, and the sources and methods of case ascertainment and control selection. Give the rationale for the choice of cases and controls	*n/a*	*n/a*	*n/a*
		(b) *Cohort study*—For matched studies, give matching criteria and number of exposed and unexposed	☐	☒	☐
		*Case-control study*—For matched studies, give matching criteria and the number of controls per case	*n/a*	*n/a*	*n/a*
Variables	7	Clearly define all outcomes, exposures, predictors, potential confounders, and effect modifiers. Give diagnostic criteria, if applicable	☐	☒	☒
Data sources/measurement	8*	For each variable of interest, give sources of data and details of methods of assessment (measurement). Describe comparability of assessment methods if there is more than one group	☐	☒	☒
Bias	9	Describe any efforts to address potential sources of bias	☐	☒	☐
Study size	10	Explain how the study size was arrived at	☐	☒	☐
Quantitative variables	11	Explain how quantitative variables were handled in the analyses. If applicable, describe which groupings were chosen and why	*n/a*	*n/a*	*n/a*
Statistical methods	12	(a) Describe all statistical methods, including those used to control for confounding	☐	☒	☒
		(b) Describe any methods used to examine subgroups and interactions	☐	☒	☐
		(c) Explain how missing data were addressed	☐	☐	☐
		(d) *Cohort study*—If applicable, explain how loss to follow-up was addressed	☐	☒	☐
		*Case-control study*—If applicable, explain how matching of cases and controls was addressed	*n/a*	*n/a*	*n/a*
		(e) Describe any sensitivity analyses	☐	☒	☐
**Results**					
Participants	13*	(a) Report numbers of individuals at each stage of study—e.g., numbers potentially eligible, examined for eligibility, confirmed eligible, included in the study, completing follow-up, and analysed	☒	☒	☒
		(b) Give reasons for non-participation at each stage	☐	☐	☐
		(c) Consider use of a flow diagram	☐	☐	☐
Descriptive data	14*	(a) Give characteristics of study participants (e.g., demographic, clinical, social) and information on exposures and potential confounders	☒	☒	☒
		(b) Indicate number of participants with missing data for each variable of interest	☐	☒	☐
		(c) *Cohort study*—Summarise follow-up time (e.g., average and total amount)	☐	☒	☒
Outcome data	15*	*Cohort study*—Report numbers of outcome events or summary measures over time	☒	☒	☒
		*Case-control study—*Report numbers in each exposure category, or summary measures of exposure	*n/a*	*n/a*	*n/a*
Main results	16	(*a*) Give unadjusted estimates and, if applicable, confounder-adjusted estimates and their precision (e.g., 95% confidence interval). Make clear which confounders were adjusted for and why they were included	☐	☒	☐
		(*b*) Report category boundaries when continuous variables were categorised	*n/a*	*n/a*	*n/a*
		(*c*) If relevant, consider translating estimates of relative risk into absolute risk for a meaningful time period	☐	☒	☐
Other analyses	17	Report other analyses done—e.g., analyses of subgroups and interactions, and sensitivity analyses	☐	☒	☐
**Discussion**					
Key results	18	Summarise key results with reference to study objectives	☒	☒	☒
Limitations	19	Discuss limitations of the study, taking into account the sources of potential bias or imprecision. Discuss both direction and magnitude of any potential bias	☒	☒	☒
Interpretation	20	Give a cautious overall interpretation of results considering objectives, limitations, multiplicity of analyses, results from similar studies, and other relevant evidence	☒	☒	☒
Generalisability	21	Discuss the generalisability (external validity) of the study results	☐	☒	☒
**Other information**					
Funding	22	Give the source of funding and the role of the funders for the present study and, if applicable, for the original study on which the present article is based	☒	☒	☐

**Table 8.** Characteristics of studies included in the systematic review on the impact of PCa on T2DM control and treatments.

Author, year, country	Study design	No. of patients	Main outcomes	Main findings
Keating, 2014, USA [81]	Cohort with propensity matching	2237 pairs of propensity matched men with PCa and T2DM who were or were not treated with ADT	The effect of ADT onT2DM control, as measured by HbA1c levels and the intensification of T2DM drug therapy.	HbA1c increased at 1 year for men treated with ADT (7.38 from 7.24 *p* value 0.04) Receipt of ADT was also associated with an increased risk of addition of T2DM medication (HR 1.20 95% CI: 1.09–1.32)
Rowbottom, 2015, Canada [83]	Cohort	30 GU Cancer patients: 26 PCa 4Bladder Ca	Change in T2DM management or hospitalisation due to T2DM in those receiving corticosteroids with chemotherapy	40% required a change in their diabetes management (*n* = 4) 20% (*n* = 2) required hospitalisations
Derweesh, 2007, USA [82]	Cohort	77 patients	To assess worsening glycaemic control in men with established T2DM after starting ADT for PCa	An increase of ≥ 10% in serum HbA1c in 15 patients (19.5%)An increase of ≥ 10% in FBG in 22 patients (28.6%)
